# Energy management of electric vehicle using a new strategy based on slap swarm optimization and differential flatness control

**DOI:** 10.1038/s41598-024-53396-3

**Published:** 2024-02-13

**Authors:** Houssam Eddine Ghadbane, Said Barkat, Ali Djerioui, Azeddine Houari, Mihai Oproescu, Nicu Bizon

**Affiliations:** 1 Electrical Engineering Laboratory of Guelma (LGEG), Electrotechnical and Automatic Engineering Department, Université, 8 Mai 1945, 24000 Guelma, Algeria; 2 Electrical Engineering Laboratory, Electrical Engineering Departement, University of M’sila, M’sila, Algeria; 3 IREENA Laboratory, Industrial Engineering Department, University of Nantes, Nantes, France; 4 Pitești University Centre, National University of Science and Technology POLITEHNICA Bucharest, 110040 Pitesti, Romania; 5 Doctoral School, Polytechnic University of Bucharest, 313 SplaiulIndependentei, 060042 Bucharest, Romania; 6 ICSI Energy Department, National Research and Development Institute for Cryogenic and Isotopic Technologies, 240050 RamnicuValcea, Romania

**Keywords:** Electrical and electronic engineering, Energy science and technology, Energy storage, Batteries, Supercapacitors

## Abstract

Optimal energy management of electric vehicles using slap swarm optimization and differential flatness control has been proposed. A battery–supercapacitor power system is adopted. Each source is connected in parallel to the DC-bus using DC–DC bidirectional converters and supplies a synchronous reluctance motor (SynRM) based drive. The proposed EMS fundamental forces lie in using a combination of complementary proprieties of two approaches, a Slap Swarm optimization Algorithm and Differential Flatness (DF). With a fast optimization mechanism, the Slap Swarm optimization algorithm allows adapting in real-time conditions the DF gains to optimize the system performances. On its side, DF uses predefined trajectories respecting the physical proprieties of the system, which is a powerful tool to guarantee the dynamic constraints of the sources when ensuring desired robust control proprieties. To check the feasibility and performance of the suggested EMS, comprehensive processor-in-the-loop co-simulations of the electric vehicle were carried out using the C2000 launchxl-f28379d DSP board. The main goal of the proposed EMS is to guarantee the DC-bus stabilization, reducing the DC-bus voltage ripples (Δv = 5 V) and the voltage overshoots 15 V (3.2%), respect the source dynamics, and satisfy the SynRM motor power demand. Furthermore, the algorithm minimizes induced harmonics by the drive (10.49%), reducing the battery current ripple by 17.15A, thereby enhancing the battery lifecycle.

## Introduction

Nowadays, the influence of the transportation system’s growth on global warming and climate change has become more and more tangible and has impacted our everyday lives^1^. According to^2^, integrating the road transport sector into the EU ETS (European Emissions Trading System) is expected to be a cost-effective CO_2_ reduction method. The transport sector accounted for roughly 27% of total EU greenhouse gas (GHG) emissions in 2017, with light commercial vehicles (LCVs) contributing to approximately 9% of EU transport GHG emissions^3^. According to the European Environment Agency (EEA), average CO_2_ emissions from new light-duty vehicles (LDVs) rose in 2017 for the first time since 2010 and continued to rise in 2018^4^. The European Commission published a proposal to outlaw the sale of petrol and diesel automobiles in the EU from 2035^5^. In this context, transportation decarbonization is primarily recognized as one of the priorities for considerably decreasing harmful emissions.

The facts presented here confirm that the automobile industry has adopted the electrified mobility vision. Several automakers have openly stated their commitment to an electric vehicle future, expecting the EV market to be poised for rapid expansion. For example, Volvo Company stated that cars with internal combustion engines have no long-term future. Volvo is firmly dedicated to becoming an all-electric vehicle manufacturer, with the transition expected to occur by 2030^6^. By 2035, General Motors intends to eliminate tailpipe emissions from new light-duty cars^7^. Following a year in which Volkswagen electric vehicle sales quadrupled, the company noticed that 2020 represented a turning point in customer mood^8^. EV conventional power system mainly involves batteries and power electronic converters^9^. It is well established that these power converters’ currents contain harmonics. The detrimental effect of current harmonics on the aging of Li-ion cells was studied based on the results of the tests conducted by the authors in^10–15^.

To provide better performance in terms of power quality, durability, and reliability, hybrid power systems (HPSs) based EVs are suggested in the literature^16–18^. HPS comprises a battery as the primary source and a supercapacitor (SC) as an auxiliary. The battery provides most of the motor power due to its high energy density. At the same time, the SC enhances the power quality by supplying the transient periods due to its high power density. The electric machine has played a significant role throughout the evolution of EVs, regardless of the precise types of EVs provided. Different electric machines have been used in the EV design; each has advantages and disadvantages. Synchronous Reluctance Motor (SynRM) has recently gained more attention in EV applications due to its increased efficiency, reduced weight, and moderate cost^19–23^. In addition, SynRM can provide torque enhancement by 34% more than the classical machines, which is suitable for the EV industry^19^. Despite the outstanding advantages described above, SynRM has several relevant disadvantages for traction applications, including torque ripple and SynRM current harmonics that affect battery lifetime (battery aging)^21^. The primary challenge is to provide an appropriate energy management strategy (EMS) that enhances the performance of the HPS and extends the battery lifecycle while considering different constraints.

However, the main focus lies in evaluating the efficacy of the chosen EMS. To attain this objective, multiple EMSs have been documented in academic literature. Broadly, these EMSs can be categorized into optimization, rule-based, and learning-based techniques, as mentioned in^24,25^. Optimization-driven approaches employ optimization theory tools to solve the issue, aiming for an optimal power load distribution across the battery–supercapacitor storage system to maximize component lifespans. The methods focused on optimization can be classified as offline (global scale) or online (real-time scale). Offline optimization EMS involves determining the best control solution for a predefined condition, like a speed profile. There are two methods for this: Direct methods encompass disciplined optimum control; Indirect methods involve approaches such as the calculus of variations^24^, Pontryagin's Maximum Principle (PMP)^26^, Pontryagin's Minimum Principle^27^, stochastic dynamic programming (SDP)^28^, and dynamic programming (DP)^29^. The real-time application of online optimization includes determining the most efficient energy distribution in a hybrid system using specified data. The cost function factors in the system's current status, operational costs, and emissions. Model predictive control (MPC)^30^, equivalent consumption minimization strategy (ECMS)^31^, and external energy maximization strategy (EEMS)^32^ fall under this category. In artificial intelligence, learning-based techniques, particularly reinforcement learning (RL)^33^ and deep learning (DL)^34^, leverage machine learning advancements. These methods have exhibited effectiveness across diverse domains, notably in image categorization, leading to widespread implementation in energy management^35,36^. However, the unavailability of databases required for training models poses a challenge, given the lack of extensive research on this emerging subject. These databases offer no assurance of compatibility with data beyond the provided training set. Rule-based strategies are management strategies based on a sequence of IF–THEN situations. This kind of management strategy can be categorized into two types: deterministic strategies, such as state machine control (SMC) strategy^27,28^, and intelligent strategies, such as fuzzy logic-based EMS^29,30^. The fundamental disadvantage of these strategies is that developing their roles requires the designer's experience, which is not always available. They also suffer from problems related to the abrupt transitions between various operating modes that present a real challenge for conventional controllers to keep the desired power quality and system stability. A linear proportional-integral (PI) based management strategy was reported in^24–35^. Authors in^37^ proposed a PI-based voltage control strategy for the common DC-bus of hybrid EVs. However, PI controller design based on linearization around an operating point suffers from robustness problems. The flatness-based management strategy (FLAT) has been widely used to manage the power flow in a multi-sources power system^38,39^. This strategy can provide excellent performance, but defining the trajectory parameters is challenging; the definition of flat outputs and the dependence on the reference model decreases these performances in case of essential parameter changes.The traditional approaches may offer restricted effectiveness. This research aims to evaluate the optimization of trajectory generation parameters utilizing a metaheuristic optimization technique known as the Salp Swarm Algorithm (SSA). The main benefits of SSA over other optimization strategies are faster solution convergence and fewer parameters with faster-resolving capability^40^. Based on the advantages of the above-mentioned optimization algorithms, this paper reports a new optimized flatness-based management strategy to manage the power flow in an EV power system while reducing battery aging. The considered hybrid power system is based on a Li-ion battery and supercapacitor as energy storage elements. The main goals of the suggested EMS are to provide DC-bus stabilization, respect the dynamics of the sources, and satisfy the SynRM motor power demand. Additionally, the algorithm allows the minimization of the effect of harmonics generated by the motor on the battery current. Thus, the main contributions can be listed as follows:

- Stabilizing the DC-bus voltage;
- Ensuring the proper use of the power flows between the battery and SC while respecting the SoC limits;
- Preventing the SynRM motor from the harmonics that impact the energy storage systems’ lifetimes;
- Reducing the battery current harmonics induced by the electric drive;
- Minimizing the battery current ripples generated by both the SynRM and PWM inverter;
- Combining different strategies to obtain the best performances.

The control algorithm and EMS for the proposed hybrid power system are validated by processor-in-the-loop (PIL) technology in Matlab/Simulink using embedded Matlab functions. The final generated control algorithm codes are embedded in a real target DSP (C2000 launchxl-f28379d). In this, the integrated DSP control algorithm manages all components of the hybrid power system (Bat-SC-SynRM), modeled in the host PC using SimPowerSystems (SPS) Toolbox.

The remainder of this work is arranged as follows: Sect. 2 presents modeling the electric traction chain's parts. Section 3 discusses the suggested EMS based on the optimal differential flatness. The results and interpretations of the co-simulations are presented in Sect. 4. The study's conclusion is presented in Sect. 5.

## System description and modeling

### Detailing the study and the proposed diagram

**Figure 1:**
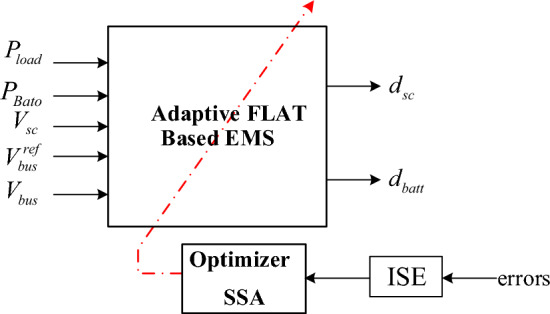
SSA-FLAT-based EMS.

Finding the specific numerical values for trajectory generation parameters presents a challenge because there is not an exact model replicating the physical system. To address this, an approach involves improving these parameters through metaheuristic optimization algorithms. The main concept involves creating random potential solutions within a confined search area. These solutions will be directed to the HPS, where their behavior will be evaluated by calculating the integral square error (ISE) between the reference and actual DC bus and supercapacitor voltage and battery and the supercapacitor current. The optimizers will adjust and update the potential solutions based on this ISE, which represents the fitness value. The optimized adaptive Flatness-based EMS is described in Figure 1.

### HPS topology

**Figure 2:**
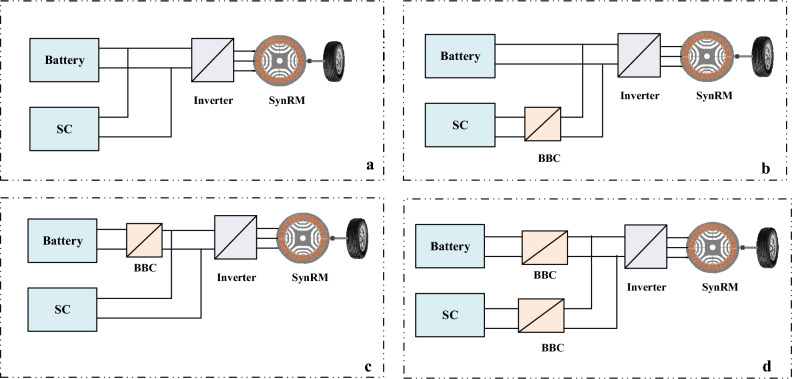
The HPS topologies: (**a**) passive topology; (**b**,**c**) semi-active topological structure ; (**d)** fully active topology.

Three hybrid power system topologies for electric vehicles, as illustrated in Fig. 2, have been commonly reported in the literature. These topologies with distinctive qualities can be categorized as passive, semi-active, and active^41^.

**Figure 3:**
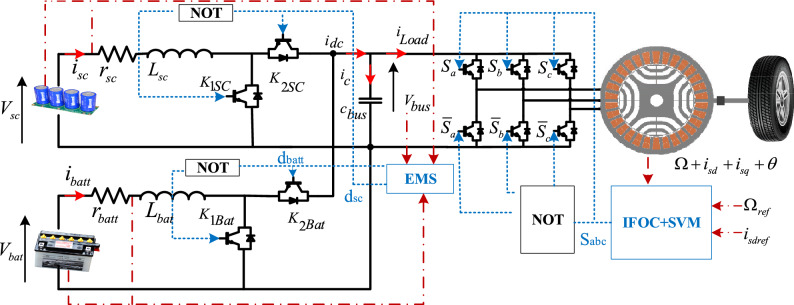
Schematic structure of the proposed EV.

The architecture of the studied HPS is based on the fully active topology, as shown in Fig. 3, Since each ESS is controlled independently, this architecture provides the maximum level of controllability. It makes it easier for the energy management plan to use the complementing traits of HPS and HES. Additionally, it supports implementing a wide range of control mechanisms^42^. The traction chain comprises two energy storage systems (battery and SC), their converters (bidirectional DC–DC), and a synchronous reluctance motor drive.

### Power system modeling

The switching and average models are the two DC–DC converters models extensively utilized in the literature. The switching model is primarily used to investigate different forms of pulse width-modulated systems in terms of power losses and harmonics. It requires a short sampling period, making simulation time-consuming; hence, the following average model was adopted in this study.

1 $$\left\{ {\begin{array}{*{20}c} {L_{bat} \frac{{di_{{_{bat} }} }}{dt}\, = V_{bat} - V_{bus} d_{batt} - r_{Bat} i_{batt} \,\,\,\,\,\,\,\,\,\,\,\,\,\,\,\,\,\,\,\,\,\,\,\,\,\,\,\,} \\ {\,\,\,L_{sc} \frac{{di_{{_{sc} }} }}{dt}\, = V_{SC} - V_{bus} d_{sc} - r_{SC} i_{SC} \,\,\,\,\,\,\,\,\,\,\,\,\,\,\,\,\,\,\,\,\,\,\,\,\,\,\,\,\,\,\,\,\,\,\,\,\,\,\,\,} \\ {\,c_{bus} \frac{{dV_{bus} }}{dt} = d_{batt} i_{bat} \, + d_{sc} i_{SC} \, - i_{Load} \,\,\,\,\,\,\,\,\,\,\,\,\,\,\,\,\,\,\,\,\,\,\,\,\,\,\,\,\,\,\,\,} \\ \end{array} } \right.$$

where *V*_*SC*_ and *V*_*bus*_ are the supercapacitor and DC-bus voltage, respectively; *r*_*SC*_ and *r*_*batt*_ denote the internal converter resistors; *L*_*SC*_ and *L*_*batt*_ are the converter inductors; *d*_*SC*_ and *d*_*batt*_ represent the duty cycle ratios; *C*_*bus*_ represents the DC-bus capacitance; ${i}_{Load}$, ${i}_{SC}$, and ${i}_{batt}$ are the load, the supercapacitor, and the battery currents, respectively.

The electromagnetic energy stored in the DC bus capacitance *E*_*bus*_ and the capacitive energy of the SC *E*_*SC*_ are given by:

2 $$E_{Bus} = 0.5\,c_{bus} V_{bus}^{2}$$

3 $$E_{SC} = 0.5\,c_{SC} V_{SC}^{2}$$

The total stored electromagnetic energy *E*_*T*_ is expressed as follows:

4 $$E_{T} = 0.5\,c_{bus} V_{bus}^{2} + 0.5\,c_{SC} V_{SC}^{2}$$

According to Fig. 1, the differential equation for power balance is^43^ :

5 $$\dot{E}_{Bus} = P_{Bato} + P_{SCo} - P_{Load}$$

The supplied powers from the sources, including converter losses, are expressed by* P*_*Bato*_ and *P*_*SCo*_ as follows:

6 $$P_{Bato} = P_{Bat} - r_{Bat} \left( {\frac{{P_{Bat} }}{{V_{Bat} }}} \right)^{2}$$

7 $$P_{SCo} = P_{SC} - r_{SC} \left( {\frac{{P_{SC} }}{{v_{SC} }}} \right)^{2}$$

where

8 $$P_{SC} = V_{SC} i_{SC} = \,i_{SC} \left( {{\raise0.7ex\hbox{${2E_{SC} }$} \!\mathord{\left/ {\vphantom {{2E_{SC} } {c_{SC} }}}\right.\kern-0pt} \!\lower0.7ex\hbox{${c_{SC} }$}}} \right)^{{{\raise0.7ex\hbox{$1$} \!\mathord{\left/ {\vphantom {1 2}}\right.\kern-0pt} \!\lower0.7ex\hbox{$2$}}}}$$

9 $$P_{Load} = V_{bus} i_{Load} = \,i_{Load} \left( {{\raise0.7ex\hbox{${2E_{Bus} }$} \!\mathord{\left/ {\vphantom {{2E_{Bus} } {c_{bus} }}}\right.\kern-0pt} \!\lower0.7ex\hbox{${c_{bus} }$}}} \right)^{{{\raise0.7ex\hbox{$1$} \!\mathord{\left/ {\vphantom {1 2}}\right.\kern-0pt} \!\lower0.7ex\hbox{$2$}}}}$$

### Synchronous reluctance motor and vehicle dynamics modeling

There are currently several main rotor structures of SynRM: solid rotor, flux barrier rotor, axially laminated rotor, and magnet-assisted rotor. The structures suitable for traction applications have flux barriers since they meet performance, robustness, cost, and manufacturing requirements^20^. The SynRM electromechanical model is presented in the (*d*-*q*) frame as follows:

10 $$\frac{d}{dt}\left[ {\begin{array}{*{20}c} \begin{gathered} i_{sd} \hfill \\ \hfill \\ \end{gathered} \\ \begin{gathered} i_{sq} \hfill \\ \hfill \\ \end{gathered} \\ \begin{gathered} \Omega \hfill \\ \hfill \\ \end{gathered} \\ \theta \\ \end{array} } \right] = \left[ {\begin{array}{*{20}c} {\frac{{ - R_{s} }}{{L_{d} }}i_{sd} + \frac{{pL_{q} }}{{L_{d} }}i_{sq} \Omega } \\ {\frac{{ - R_{s} }}{{L_{q} }}i_{sq} - \frac{{pL_{d} }}{{L_{q} }}i_{sd} \Omega } \\ \begin{gathered} \frac{{3p(L_{d} - L_{q} )i_{sd} i_{sq} }}{2J} - \frac{f}{J}\Omega - T_{L} \hfill \\ \,\,\,\,\,\,\,\,\,\,\,\,\,\,\,\,\,\,\,\,\,\,\,\,\,p\Omega \hfill \\ \end{gathered} \\ \end{array} } \right] + \left[ {\begin{array}{*{20}c} \begin{gathered} \frac{1}{{L_{d} }} \hfill \\ \hfill \\ \end{gathered} \\ \begin{gathered} 0 \hfill \\ \hfill \\ \end{gathered} \\ \begin{gathered} 0 \hfill \\ \hfill \\ \end{gathered} \\ 0 \\ \end{array} \,\,\begin{array}{*{20}c} \begin{gathered} 0 \hfill \\ \hfill \\ \end{gathered} \\ \begin{gathered} \frac{1}{{L_{q} }} \hfill \\ \hfill \\ \end{gathered} \\ \begin{gathered} 0 \hfill \\ \hfill \\ \end{gathered} \\ 0 \\ \end{array} } \right]\left[ {\begin{array}{*{20}c} {v_{sd} } \\ {v_{sq} } \\ \end{array} } \right]$$

where *v*_*sq*_ and *v*_*sd*_ are the stator's quadrature and direct axis voltages, respectively. *i*_*sq*_ and *i*_*sd*_ represent their corresponding currents. The *L*_*d*_ and *L*_*q*_ are the *d*-*q* magnetizing inductances. *Ω* represents the mechanical rotation speed, *R*_*s*_ represents the stator resistance, *p* denotes the number of pair poles, *T*_*L*_, is the rotor's torque load, *f* and *J* represent the viscous friction coefficient, and inertia moment.

The equation representing the dynamics of an electric motor is formulated as follows^18^:

11 $$T_{e} - T_{Load} = J_{ve} \frac{d\Omega }{{dt}}$$

The torque of the load is provided by:

12 $$T_{Load} = \frac{{T_{Lwheel} }}{i} = \frac{r}{i}\left[ {(m_{v} \,g\,\mu \,sign(v)) + (0.5\,\gamma \,C_{d} \,A_{f} \,(v\underline { + } v_{w} )^{2} \,)\underline { + } (m_{v} \,g\,\sin \,\alpha \,)\underline { + } (k_{m} \,m_{v} \frac{dv}{{dt}})} \right]$$

where ${T}_{Lwheel },i ,$ and $r$ represent the load torque on the wheels, the transmission ratio, and the wheel radius, $v$ and ${v}_{w}$ are the vehicle and wind speed, respectively, $\alpha$ represents the angle of the slop, ${m}_{v}$ is vehicle masse, *µ*,${k}_{m}$ , and ${C}_{d}$ are the tire rolling resistance, the rotational inertia, and aerodynamic drag coefficient, respectively,$\gamma$ and ${A}_{f }$ represent the air density and the frontal vehicle area. Respectively, g represents the earth's gravity.

## Classification and comparative analysis of the energy management approach

**Figure 4:**
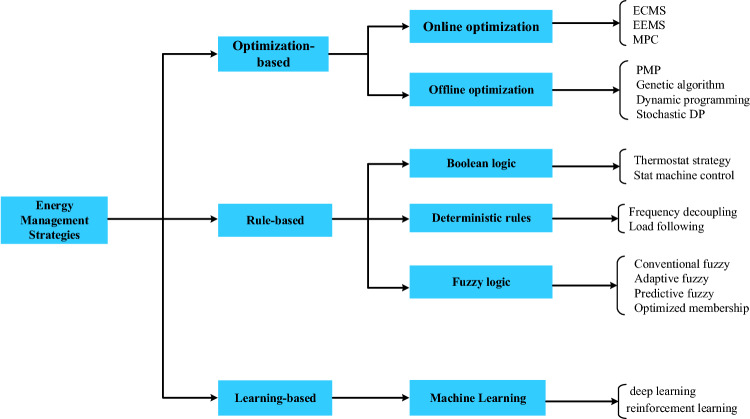
Classification of EMS of HPS.

EMSs can be partitioned into three main methods: optimization-based methods, rule-based methods, or more recently, learning-based methods, as depicted in Fig. 4.

**Table 1:** Comparative analysis of EMS^47^.

EM strategy	Advantages	Limitations	Refs.
Deterministic rule-based	Simple, reliable, and robust, low computation complexity, easy to implement	Not adaptive and poor parametric calibration	^48^
Frequency separation	Simple and easy implementation	Not robust, filter design is difficult	^49^
Fuzzy logic Control	Robust and good with model uncertainties and state variations, real-time implementable	Dependent on membership functions, optimal control is not guaranteed	^50^
Dynamic programming	Global optimal solution is found, optimal control, easy to solve nonlinear optimization problems	Not real-time implementable requires heavy computational burden	^51^
Pontryagin's principle	Easy to adapt and simple to implement, no additional controllers are required	The computation burden is heavy	^52,53^
Instantaneous optimization	The optimal value is found at each instant	Not guaranteed to be optimal,	^54,55^
Model predictive control	Has potential for real-time implementations; Easy to handle constraints directly in the design procedure	Model accuracy may be compromised by using a linearized model May need large memory for heavy computations	^30^
Reinforcement Learning (RL)	RL can adapt to unknown environments; DL handles complex nonlinear relationships	RL requires significant exploration time. DL might lack explainability and require extensive data	^56^
Neural network	It is more robust to new information, real time implementable	Large amount of training data is required; stability is not guaranteed	^57^

### The suggested EMS

The suggested EMS aims to control the motor speed effectively and stabilize the voltage of the DC bus. Indirect Field Oriented Control (IFOC) is used to control the motor speed. This strategy provides the d-q voltage references for the State Space Vector PWM (SVPWM) system. Hybridization between the differential flatness theory and metaheuristic optimization is used to control the bus voltage optimally.

### SynRM drive IFOC design

**Figure 5:**
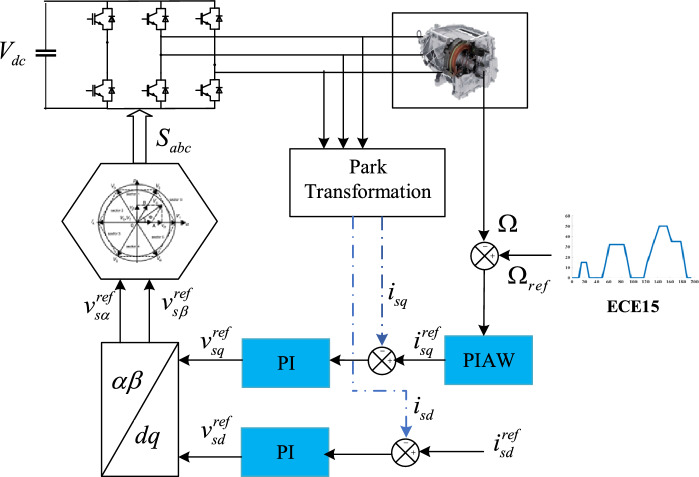
Diagram of SynRM Field-oriented control.

The SynRM’s Indirect Field Oriented Control (IFOC), as shown in Fig. 5, comprises three control loops: an external speed control loop, an internal current control loop for ${i}_{sd}$ and another loop for ${i}_{sq}$. The first loop output generates the quadrature reference current, which is compared to the current value resulting from the current measurements. Their error is applied to the input of the *i*_*sq*_ current controller. Similarly, there is a current regulation loop of direct current *i*_*sd*_. The outputs of the two internal loops *i*_*sq*_ and* i*_*sd*_ are applied to a decoupling block generating the reference voltages ${v}_{sd}^{ref}$ and ${v}_{sq}^{ref}$. By passing from the reference (*d*-*q*) to the reference (*α*,*β*), the two voltages reference ${v}_{s\alpha }^{ref}$, ${v}_{s\beta }^{ref}$ required by the Space Vector Modulation (SVM) block.

### The proposed optimal differential flatness-based EMS

**Figure 6:**
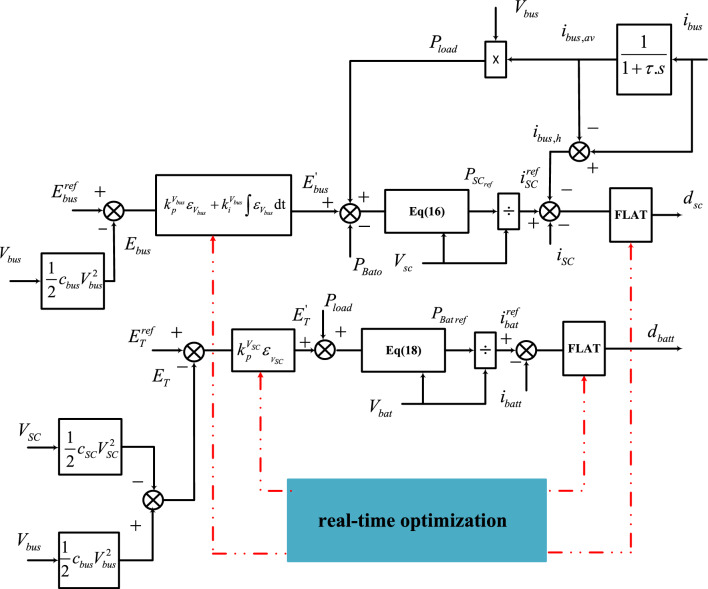
Proposed optimal differential flatness-based EMS.

The proposed EMS is based on optimal differential flatness. It is divided into two parts: a lower-level controller and a higher-level controller. The higher-level controller generates the power reference for each source. The power references created by the inverse dynamic in Eq. (16) and Eq. (18) are divided by the measured supercapacitor and battery voltages to generate the reference currents for the supercapacitor and battery converters. The lower-level controller adjusts for DC-bus current variations and compensates for the DC-bus current harmonics using a supercapacitor, which results in improved energy quality and battery lifecycle enhancement. Figure 6, illustrates the operational control structure.

#### Energy regulation

In the considered HPS, the flat model is expressed by its flat output $y={[{y}_{1} {y}_{2}]}^{T}$, control variable $u={[{u}_{1} {u}_{2}]}^{T}$, and state variable $x={[{x}_{1} {x}_{2}]}^{T}$,

Where:

13 $$y = \left[ {\begin{array}{*{20}c} {E_{Bus} } \\ {E_{T} } \\ \end{array} } \right],u = \left[ {\begin{array}{*{20}c} {P_{SC}^{ref} } \\ {P_{Bat}^{ref} } \\ \end{array} } \right],x = \left[ {\begin{array}{*{20}c} {V_{bus} } \\ {V_{SC} } \\ \end{array} } \right]$$

From Eq. (2), the state variable ${x}_{1}$ representing *V*_*bus*_ can be expressed as follows:

14 $$x_{1} = \left( {{\raise0.7ex\hbox{${2y_{1} }$} \!\mathord{\left/ {\vphantom {{2y_{1} } {c_{bus} }}}\right.\kern-0pt} \!\lower0.7ex\hbox{${c_{bus} }$}}} \right)\,^{{{\raise0.7ex\hbox{$1$} \!\mathord{\left/ {\vphantom {1 2}}\right.\kern-0pt} \!\lower0.7ex\hbox{$2$}}}} = \lambda_{1} (y_{1} )$$

From Eq. (4), the state variable ${x}_{2}$ representing *V*_*SC*_ can be expressed as follows:

15 $$x_{2} = \left( {{\raise0.7ex\hbox{${2(y_{2} - y_{1} )}$} \!\mathord{\left/ {\vphantom {{2(y_{2} - y_{1} )} {c_{SC} }}}\right.\kern-0pt} \!\lower0.7ex\hbox{${c_{SC} }$}}} \right)^{{{\raise0.7ex\hbox{$1$} \!\mathord{\left/ {\vphantom {1 2}}\right.\kern-0pt} \!\lower0.7ex\hbox{$2$}}}} = \lambda_{2} (y_{1} ,y_{2} )$$

The SC power reference* P*_*SCref*_, considered the first input control variable, ${u}_{1}$, is derived from Eqs. 5, 7, and 9.

16 $$u_{1} = 2P_{SC\,\max } \left[ {1 - \left( {1 - \frac{{\dot{y}_{1} + \sqrt {\frac{{2y_{1} }}{{c_{bus} }}\,} \,i_{Load} - P_{Bato} }}{{P_{SC\,\max } }}} \right)^{{{\raise0.7ex\hbox{$1$} \!\mathord{\left/ {\vphantom {1 2}}\right.\kern-0pt} \!\lower0.7ex\hbox{$2$}}}} } \right] = \vartheta_{1} (y_{1} ,\dot{y}_{1} ) = P_{{SC_{ref} }}$$

with *P*_*SCmax*_ is the SC converter's maximum power limit. It can be defined as

17 $$P_{SC\,\max } = V_{SC}^{2} /4r_{SC}$$

Concerning the second input, the power reference of the battery *P*_*Battref*_ is calculated from Eqs. 5, 6, and 9 as follows:

18 $$u_{2} = 2P_{Bat\,\,\max } \left[ {1 - \left( {1 - \frac{{\dot{y}_{2} + \sqrt {\frac{{2y_{1} }}{{c_{bus} }}\,} \,i_{Load} }}{{P_{\,Bat\,\,\max } }}} \right)^{{{\raise0.7ex\hbox{$1$} \!\mathord{\left/ {\vphantom {1 2}}\right.\kern-0pt} \!\lower0.7ex\hbox{$2$}}}} } \right] = \vartheta_{2} (y_{1} ,\dot{y}_{2} ) = P_{Bat\,ref}$$

where *P*_*Battmax*_ is defined as the maximum limiting power from the battery converter.

19 $$P_{Bat\,\max } = V_{bat}^{2} /4r_{Bat}$$

Above all, the flat output (${y}_{1}={E}_{bus}$) is the most crucial variable to control. For this, a classical PI controller is used to ensure that this flat variable is under control. Assuming the SC control loop is significantly faster than the battery control loop^44^, the DC-bus power stated in Eq. (7) can be approximated as:

20 $$\dot{E}_{Bus} = P_{SCo}$$

The transfer function is represented as a straightforward integrator. A PI regulator is used to control this part^44^. Recognizing that ${y}_{1}={E}_{bus}$, as shown below:

21 $$\dot{y}_{1} = \frac{1}{s}(k_{p}^{{V_{bus} }} + \frac{{k_{i}^{Vbus} }}{s})\left( {y_{1} - y_{1 - ref} } \right)$$

where ${k}_{p}^{{V}_{bus}}$,${k}_{i}^{{V}_{bus}}$ are the integral and the proportional gains chosen so that the closed loop characteristic polynomial is expressed as follows:

22 $$p(s) = s^{2} + \lambda_{1} s + \lambda_{0}$$

Clearly, the error $e_{1} = y_{1} - y_{1 - ref}$ meets:

23 $$\ddot{e}_{1} + k_{p}^{{V_{bus} }} \dot{e}_{1} + k_{i}^{Vbus} e_{1} = 0$$

By matching the characteristic polynomial $p(s)$ to a desired one $p_{des} (s)$, given by Eq. (22), with pre-specified root positions, an adequate choice of controller parameters can be calculated by Eq. (25) and Eq. (26).

24 $$p_{des} (s) = s^{2} + 2\xi \omega_{n} s + \omega_{n}^{2}$$

25 $$k_{p}^{{V_{bus} }} = 2\xi \omega_{n}$$

26 $$k_{i}^{{V_{bus} }} = \omega_{n}^{2}$$

where ${\omega }_{n}$ represents the natural frequency, and ξ represents the dumping ratio.

The SC energy control loop depends on total energy management. A linearizing feedback control rule is used to achieve an exponential asymptotic tracking of the trajectory^44^ as follows:

27 $$\left( {\dot{y}_{2} - \dot{y}_{2 - ref} } \right) + k_{p}^{{V_{SC} }} \left( {y_{2} - y_{2 - ref} } \right) = 0$$

28 $$k_{p}^{{V_{SC} }} = \omega_{n}^{2}$$

where ${k}_{p}^{{V}_{SC}}$ is the proportional gain of the SC voltage controller.

#### Current control

The flat output ${y=[{y}_{3} {y}_{4}]}^{T}$, control variable ${u=[{u}_{3} {u}_{4}]}^{T}$, and state variable ${x=[{x}_{3} {x}_{4}]}^{T}$ may be written as follows:

29 $$y = \left[ {\begin{array}{*{20}c} {i_{bat} } \\ {i_{SC} } \\ \end{array} } \right],u = \left[ {\begin{array}{*{20}c} {d_{bat} } \\ {d_{sc} } \\ \end{array} } \right],x = \left[ {\begin{array}{*{20}c} {i_{bat} } \\ {i_{SC} } \\ \end{array} } \right]$$

The control vector variables ${u}_{3}$, ${u}_{4}$ are evaluated from Eq. (1) and Eq. (29) as follows:

30 $$u_{3} = {\raise0.7ex\hbox{$1$} \!\mathord{\left/ {\vphantom {1 {V_{bus} }}}\right.\kern-0pt} \!\lower0.7ex\hbox{${V_{bus} }$}}\left( {L_{bat} \dot{y}_{3} - V_{bat} + r_{batt} y_{3} } \right) = \upsilon_{1} (y_{3} ,\dot{y}_{3} ) = d_{bat}$$

31 $$u_{4} = {\raise0.7ex\hbox{$1$} \!\mathord{\left/ {\vphantom {1 {V_{bus} }}}\right.\kern-0pt} \!\lower0.7ex\hbox{${V_{bus} }$}}\left( {L_{sc} \dot{y}_{4} - V_{SC} + r_{SC} y_{4} } \right) = \upsilon_{2} (y_{4} ,\dot{y}_{4} ) = d_{sc}$$

The first current control law, ${y}_{3-ref}$, defines the set-point for the battery current. The closed-loop control law is written as follows^37,45^:

32 $$\left( {\dot{y}_{3} - \dot{y}_{3 - ref} } \right) + k_{p}^{bat} \left( {y_{3} - y_{3 - ref} } \right) + k_{i}^{bat} \int {\left( {y_{3} - y_{3 - ref} } \right)dt = 0}$$

where ${k}_{i}^{bat}$ and ${k}_{p}^{bat}$ represent the controller's parameters. Let us consider the following desired dynamic polynomial^46^:

33 $$p_{1} (s) = s^{2} + 2\xi_{i1} \omega_{ni1} s + \omega_{ni1}^{2}$$

By matching the derivative of Eq. (32) with the desired dynamic polynomial ${p}_{1}(s)$ with predetermined root locations, the appropriate controller parameters are expressed by:

34 $$k_{p}^{bat} = \xi_{i1} \omega_{ni1}$$

35 $$k_{i}^{bat} = \omega_{ni1}^{2}$$

The second current control law based on feedback regulation is written as follows:

36 $$\left( {\dot{y}_{4} - \dot{y}_{4 - ref} } \right) + k_{p}^{SC} \left( {y_{4} - y_{4 - ref} } \right) + k_{i}^{SC} \int {\left( {y_{4} - y_{4 - ref} } \right)dt = 0}$$

By matching the derivative of Eq. (36) with the following desired characteristic polynomial $p_{2} (s)$ with pre-specified root positions, the controller parameters can be obtained as in Eq. (38) and Eq. (39).

37 $$p_{2} (s) = s^{2} + 2\xi_{i2} \omega_{ni2} s + \omega_{ni2}^{2}$$

38 $$k_{p}^{SC} = 2\xi_{i2} \omega_{ni2}$$

39 $$k_{i}^{SC} = \omega_{ni2}^{2}$$

where ${y}_{3-ref}$ and ${y}_{4-ref}$ are the required inductor current references;${k}_{i}^{sc}$,${k}_{i}^{bat}$ are the integral gains of the supercapacitor and battery current controllers, respectively.${k}_{p}^{sc}$ and ${k}_{p}^{bat}$ represent the proportional gains of the supercapacitor and the battery current controllers, respectively. ${\omega }_{ni1}$,${\omega }_{ni2}$ and ξ_i1_, ξ_i2_ represent the natural frequencies and the dumping factors, respectively.

Salp Swarm Algorithm:

Slap swarm algorithm (SSA) was proposed by Mirjalili in^32^ and inspired by the behavior of slaps in the ocean. It is distinguished primarily by its high precision and quick convergence capabilities. There are two sorts of agents in the agent set: leaders and followers, and their movements can be described as:

40 $$LP(n) = \left\{ {\begin{array}{*{20}c} {FP(n) + c_{1} \left( {\left( {ub - lb} \right)c_{2} + lb} \right)\,\,\,\,\,\,\,if\,\,c_{3} \ge {\raise0.7ex\hbox{$1$} \!\mathord{\left/ {\vphantom {1 2}}\right.\kern-0pt} \!\lower0.7ex\hbox{$2$}}} \\ {FP(n) - c_{1} \left( {\left( {ub - lb} \right)c_{2} + lb} \right)\,\,\,\,\,\,\,if\,\,c_{3} < {\raise0.7ex\hbox{$1$} \!\mathord{\left/ {\vphantom {1 2}}\right.\kern-0pt} \!\lower0.7ex\hbox{$2$}}} \\ \end{array} } \right.$$

where $LP(n)$ and $FP(n)$ are the leader and food position at iteration (*n*), respectively, and c_2_ and c_3_ are arbitrary variables [0,1].${u}_{b}$ and ${l}_{b}$ are the higher and lower search space boundaries, respectively. The coefficient c_1_ is the most significant parameter that affects the algorithm performance since it balances exploration and exploitation. It is described as:

41 $$c_{1} = 2e^{{ - \left( {{\raise0.7ex\hbox{${4n}$} \!\mathord{\left/ {\vphantom {{4n} {N_{\max } }}}\right.\kern-0pt} \!\lower0.7ex\hbox{${N_{\max } }$}}} \right)^{2} }}$$

where ${N}_{max}$ is the maximum number of iterations.

The movement of the follower can be expressed as:

42 $$FP_{i} (n) = {\raise0.7ex\hbox{$1$} \!\mathord{\left/ {\vphantom {1 2}}\right.\kern-0pt} \!\lower0.7ex\hbox{$2$}}\left( {FP_{i} \left( {n - 1} \right) + FP_{i - 1} \left( n \right)} \right)$$

where ${FP}_{i}(n)$ is the position of the *i*-th follower. This last one updates its position based on its position and the position of the previous salp. The objectives are attenuating the battery current harmonics and DC voltage ripples, reducing the voltage overshoots, and ensuring the stable operation of the HPS. Thun, the cost function can be formulated as the integral square error (ISE) as:

43 $$f = \min \left( {\int\limits_{0}^{t} {\sqrt \varepsilon dt} } \right)$$

For the hybrid power systems (HPSs), there are four errors written as follows:

44 $$\left\{ {\begin{array}{*{20}c} {\varepsilon_{{V_{bus} }} = V_{bus}^{ref} - V_{bus} \,\,\,\,\,\,\,} \\ {\varepsilon_{{_{{V_{SC} }} }} = V_{SC}^{ref} - V_{SC} \,\,\,\,\,\,\,} \\ {\varepsilon_{{_{ibatt} }} = i_{bat}^{ref} - i_{batt} \,\,\,\,\,\,\,\,\,} \\ {\,\varepsilon_{{_{{i_{SC} }} }} = (i_{SC}^{ref} - i_{h} ) - i_{SC} } \\ \end{array} } \right.$$

where ${\varepsilon }_{Vbus}$, ${\varepsilon }_{SC}$ are the DC-bus and the supercapacitor voltage errors, respectively, ${\varepsilon }_{ibatt}$ and ${\varepsilon }_{iSC}$ represent the battery and the supercapacitor current errors.${i}_{SC}^{ref}$ and ${i}_{SC}$ represent the SC's reference and measured currents; ${i}_{bat}^{ref}$ and ${i}_{batt}$ are the battery's reference and measured currents, respectively; ${V}_{bus}^{ref}$ and ${V}_{bus}$ denotes the DC bus's reference and measured voltages, respectively; ${V}_{SC}^{ref}$ and ${V}_{SC}$ are the SC's reference and measured voltages; and ${i}_{h}$ is the harmonic current.

Relying on the adopted cost function representing the ISE, the SSA calculates the necessary controllers’ parameters ${k}_{p}^{{V}_{bus}}$,${k}_{i}^{{V}_{bus}}$,${k}_{p}^{{V}_{SC}}$,${k}_{i}^{sc}$ ,${k}_{p}^{sc}$ ,${k}_{i}^{bat}$ , ${k}_{p}^{bat}$.

### PIL implementation technique description and performing steps

**Figure 7:**
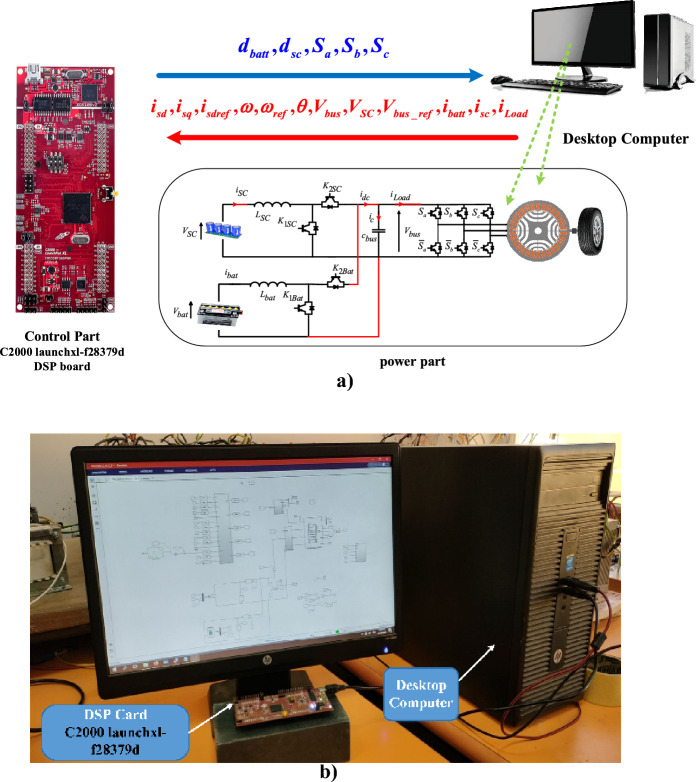
PIL co-simulation strategy: (**a**) PIL scheme, (**b**) PIL platform.

The PIL co-simulation technique allows the verification and validation of the proposed control algorithms by generating code onto the embedded processor core and running these algorithms in a real environment based on the C2000 launchxl-f28379d DSP board. During PIL co-simulation, the implemented control algorithm is linked to a computer on which the physical system model is carried out. Subsequently, it is possible to evaluate the performance of the system in order to assess and improve some essential factors such as storage capacity, code size, and execution of the algorithm according to the required time. As indicated in Fig. 7, during the prototyping of the PIL, based on a fixed simulation time, the power part of the power system is simulated in the Matlab/Simulink platform. At each step, the C2000 launchxl-f28379d DSP board receives the signals from the computer, implements control algorithms, and sends the control commands back to the computer to control the power system. At this point, a PIL co-simulation cycle is performed. The data exchange between the computer and the DSP board is synchronized using the serial communication of the DSP board. To perform the PIL, the following steps are needed to be carried out:

- Connecting the C2000 launchxl-f28379d DSP board to the computer,
- Tuning several settings and configuration parameters from the select hardware implementation tab found on Matlab/Simulink,
- Selecting the appropriate hardware port using the defined DSP board under the Hardware implementation,
- Selecting target hardware resources and selecting the correct device name of the adopted DSP board again,
- Choosing the external mode to set up the serial communication.

After that, a configuration of the PIL procedure should be performed based on multiple line codes in the command window of the Matlab software as follows:

- Calling of the system model,
- Setting the com port number,
- Defining the board rates, which represent how fast the computer and the DSP board will communicate,
- Enabling the serial communication for the PIL co-simulation,
- Generating the PIL model that will be used for the PIL procedure.

A trial implementation in a real-time interface (RTI) utilizing a DSpace card can be conducted to enhance the proposed energy management in the electric vehicle (EV) system. This experimental evaluation aims to assess the performance of the enhanced energy management system.

The experimental implementation in a real-time interface (RTI) using a Dspace card for the proposed energy management optimization in an electric car system is a promising avenue for advancing research in sustainable transportation. By leveraging the computational capabilities of a Dspace card within the vehicle's infrastructure, the energy management system can dynamically optimize power distribution among various components, such as the battery, motor, and auxiliary systems, in real-time.

## Results and discussion

### Simulation part results

In this section, MATLAB-Simulink is utilized to construct the HPS model and validate the suggested EMS. The HPS is simulated employing the urban drive cycle ECE-15, where the HPS and SSA parameters are summarized in Table 1,2, respectively.

**Table 2:** HPS parameters.

Parameters	Value
$r_{batt}$ Ω	0.1
$r_{sc}$ Ω	0.1
$L_{bat}$ mH	2
$L_{sc}$ mH	2
$V_{bus}^{ref}$ V	500
$V_{SC}^{ref}$ V	200
$V_{bat}^{ref}$ V	100
$c_{SC}$ F	80
$c_{bat}$ Ah	340
$c_{bus}$ µF	2000

**Figure 8:**
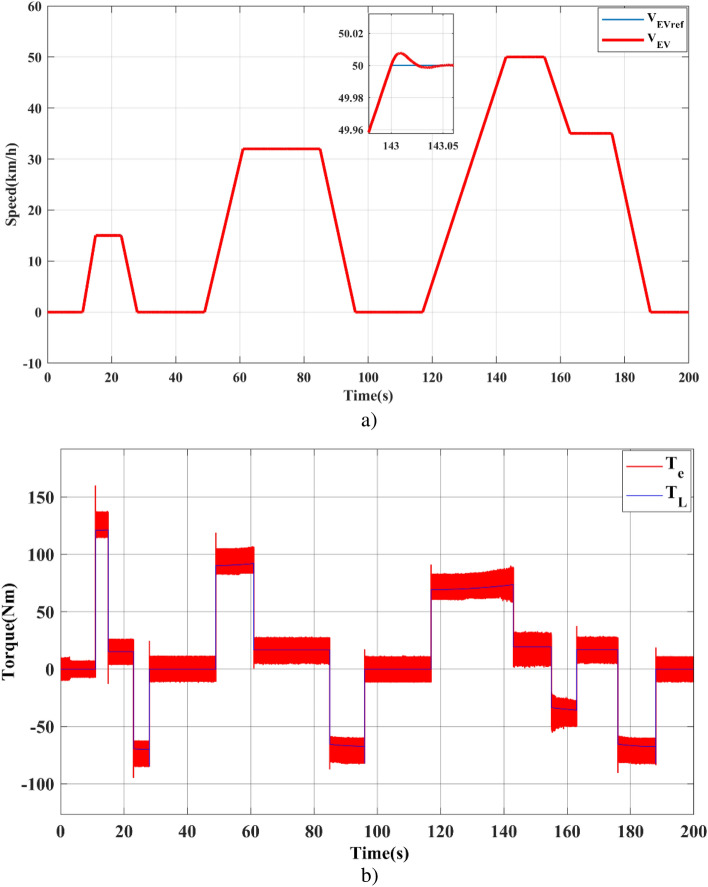
EV traction side simulation results: (**a**) EV linear speed, (**b**) EV load torque (TL) and SynRM electromagnetic torque (Te).

Notwithstanding the change in the driving cycle, the EV speed response is shown in Fig. 8.a, showing a good follow-up. The torque curve in Fig. 8.b, shows that the motor creates max torque when the vehicle's velocity attains the reference path. When it reaches a steady state, the motor provides lower torque, only enough to compensate for total load torque.

**Figure 9:**
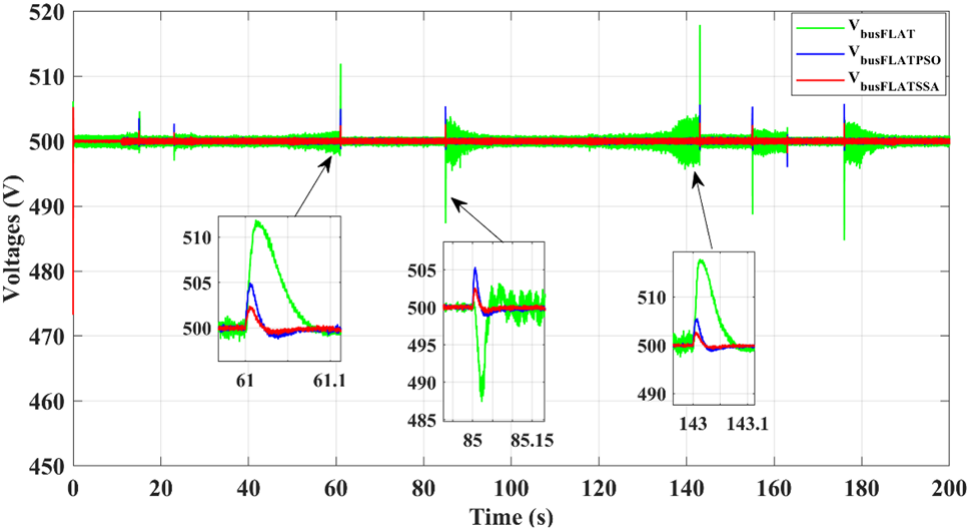
DC-bus voltage.

The suggested energy management technique can stabilize the DC-bus voltage immediately, as depicted in Fig. 9, despite the significant load power variations. Compared to the classical differential flatness and the PSO differential flatness, the proposed EMS based on the SSA differential flatness reduces DC-bus voltage ripples and overshoots. Indeed, for a maximum load step of 21 kW, the DC-bus voltage overshoot is minimized by 15V (3.2%) and 3V (0.6%) compared to the classical differential flatness and PSO differential flatness.

**Figure 10:**
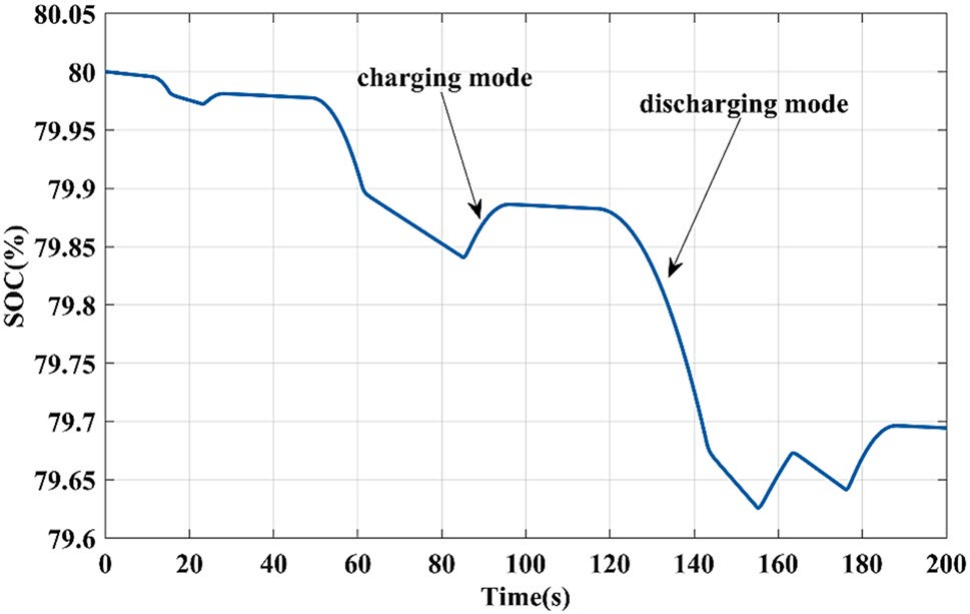
Battery SoC (%).

The battery provides most of the load power to the motor during the acceleration phases, which causes the battery SOC to decrease. It receives energy during deceleration phases when the motor torque is negative, thus increasing the SOC battery, as shown in Fig. 10.

**Figure 11:**
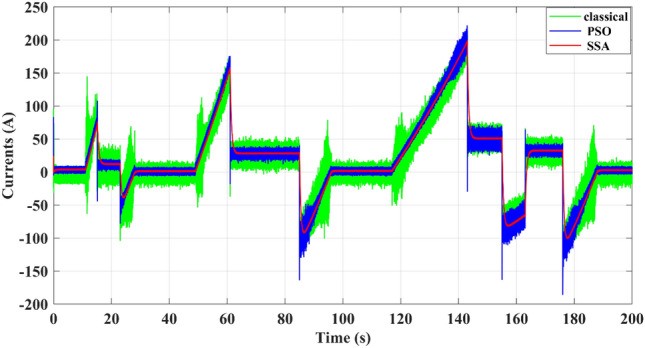
Battery Current.

From Fig. 11, which depicts the battery current, the proposed EMS can improve the power quality by reducing the current ripple, which is the main cause of battery aging, thus enhancing the battery life cycle.

Table 3 provides the comparison study results between the proposed EMS based on optimal adaptive and classical differential flatness strategies. In this table, the battery current ripple differences ΔI_1_ = ΔI_C_FLAT_–ΔI_SSA_FLAT_ and ΔI_2_ = ΔI_PSO_FLAT–_ΔI_SSA_FLAT_ are evaluated, where ΔI_C_FLAT_ and ΔI_SSA_FLAT_, ΔI_PSO_FLAT_ are the battery current ripple at the periods (P_n_) for classical FLATand SSA_FLAT, PSO_FLAT, respectively. The battery current ripple is minimized by 15.5A for a maximum load step of 21kW.

**Table 3:** Optimizer parameters.

gains	${k}_{p}^{{V}_{bus}}$	${k}_{i}^{{V}_{bus}}$	${k}_{p}^{{V}_{SC}}$	${k}_{i}^{sc}$	${k}_{p}^{sc}$	${k}_{i}^{bat}$	${k}_{p}^{bat}$
Lower bound (× 10)	100^2	418.879	0.01	8.7730 $\times$ 10^4^	8.3576	9.7478 $\times$ 10^3^	27.8253
Upper bound (× 0.5)	100^2	418.879	0.01	8.7730 $\times$ 10^4^	8.3576	9.7478 $\times$ 10^3^	27.8253
Search Agents number = 30 Maximum number of iteration = 150							

**Figure 12:**
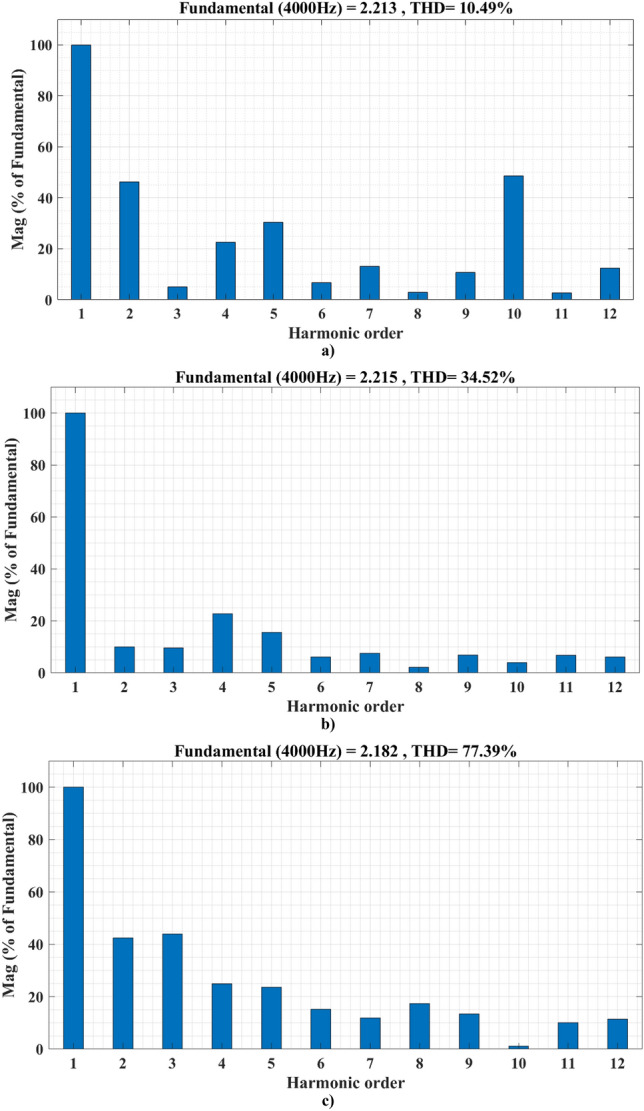
Harmonics spectrum of battery current: (**a**) for SSA differential flatness strategy; (**b**) for PSO differential flatness strategy; (**c**) for classical differential flatness strategy.

Figure 12, shows that the SSA differential flatness minimizes the battery current THD to 10.49% instead of 77.39% for the classical differential flatness strategy and 34.52 for the PSO differential flatness strategy. This finding suits the purpose of the proposed EMS, which is the reduction of battery current harmonics leading to battery lifecycle enhancement.

**Figure 13:**
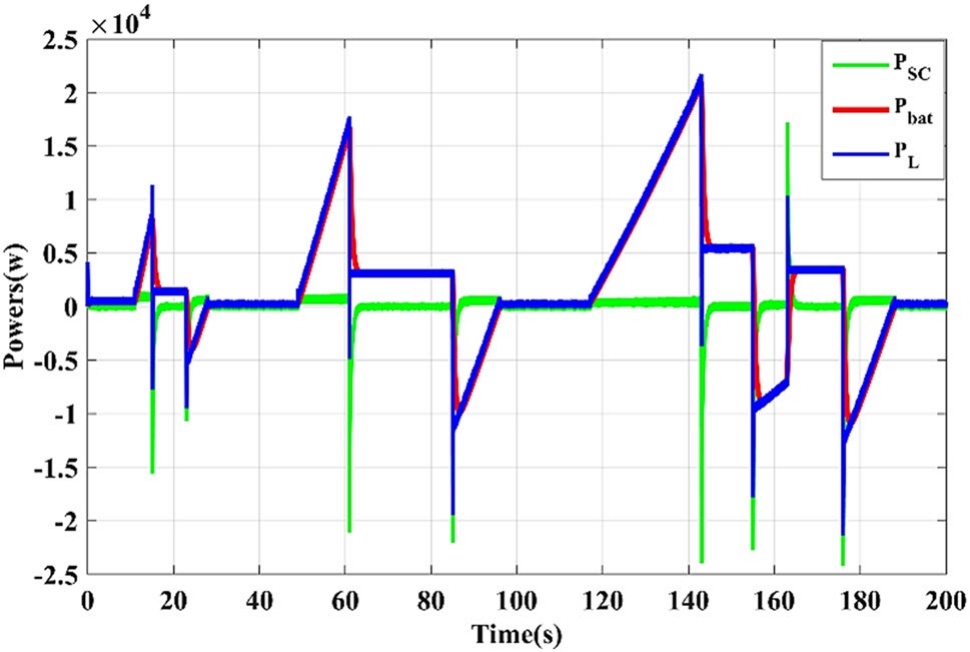
Load, battery, and SC power curves

As shown in Fig. 13, the battery supplies the motor power and absorbs it during braking periods, while the SC works to assist the battery during transient periods (acceleration and deceleration phases), which accords with the management strategy.

**Figure 14:**
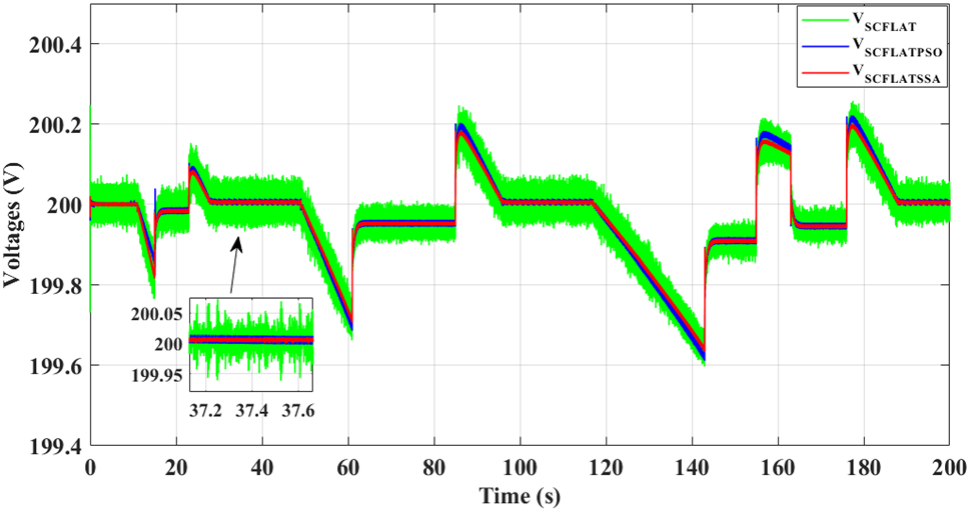
Supercapacitor voltage.

Figure 14, shows that the suggested EMS algorithm exhibits quicker supercapacitor voltage dynamics with fewer ripples than the classical differential flatness technique. The quick response of the proposed management system leads to enhancing the stability of the SC voltage.

### Co-simulation results

**Figure 15:**
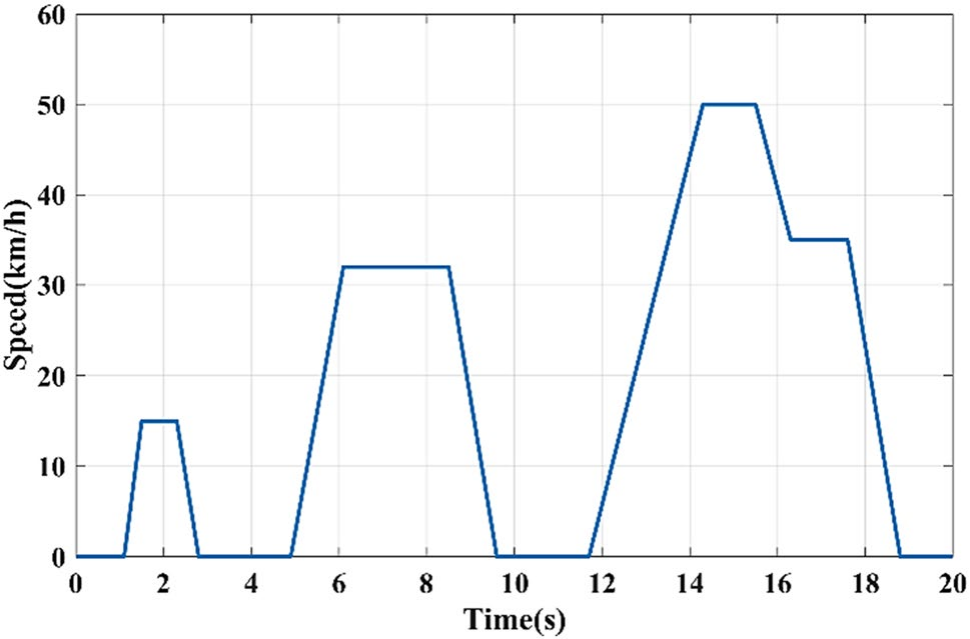
Reduced time version of the ECE-15 urban drive cycle

To approve and evaluate the performance of the suggested EMS, the system was modeled using embedded Matlab functions and co-simulated using the C2000 launchxl-f28379d DSP board through the processor-in-the-loop (PIL). The co-simulation is performed utilizing a reduced-time version of the ECE-15 urban drive cycle depicted in Fig. 15, based on the parameters listed in Table 4, Tables A1 and A4.

**Table 4:** Comparison based on battery current ripple between classical and proposed differential flatness.

Time (P_n_) (s)	P_1_ = [0,11]	P_2_ = [28,49]	P_3_ = [61,85]	P_4_ = [143,155]
ΔI_C_FLAT_ (A)	20	15	18	20
ΔI_PSO_FLAT_ (A)	10	6	8.7	15
ΔI_SSA _FLAT_ (A)	3	2.8	2.5	2.85
ΔI_1_	17	12.2	15.5	17.15
ΔI_2_	10	9	9.3	5

**Figure 16:**
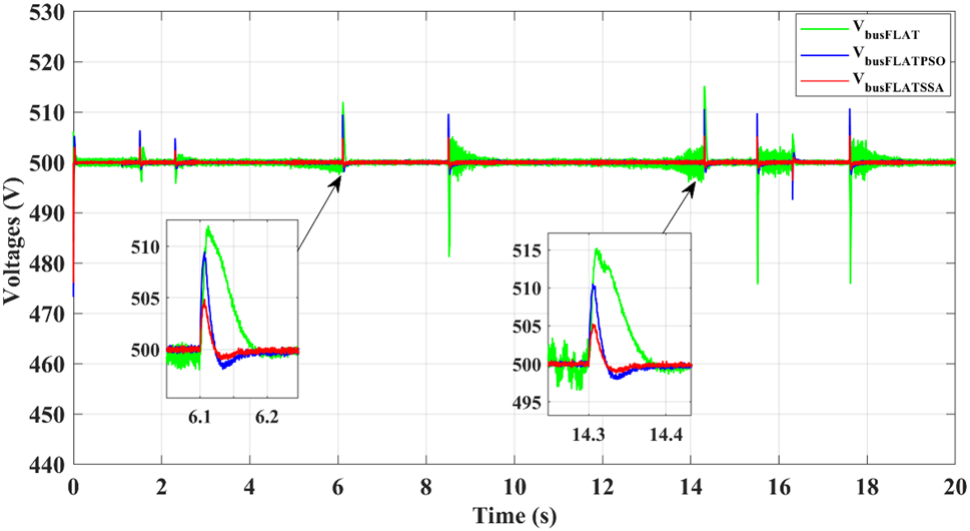
DC-bus voltage.

The proposed EMS stabilizes the bus voltage shown in Fig. 16, against load variations. Compared to classical differential FLAT, using the SSA differential flatness, the maximum overshoot voltage is 4 V instead of 10 for the PSO differential flatness and 15 V for the classical FLAT strategy at the maximum power demanded by the electric vehicle (about 21 kW). For the classical FLAT strategy, the SSA differential flatness minimizes the voltage ripple at (ΔV = 0.5 V) instead of (ΔV = 4 V).

**Figure 17:**
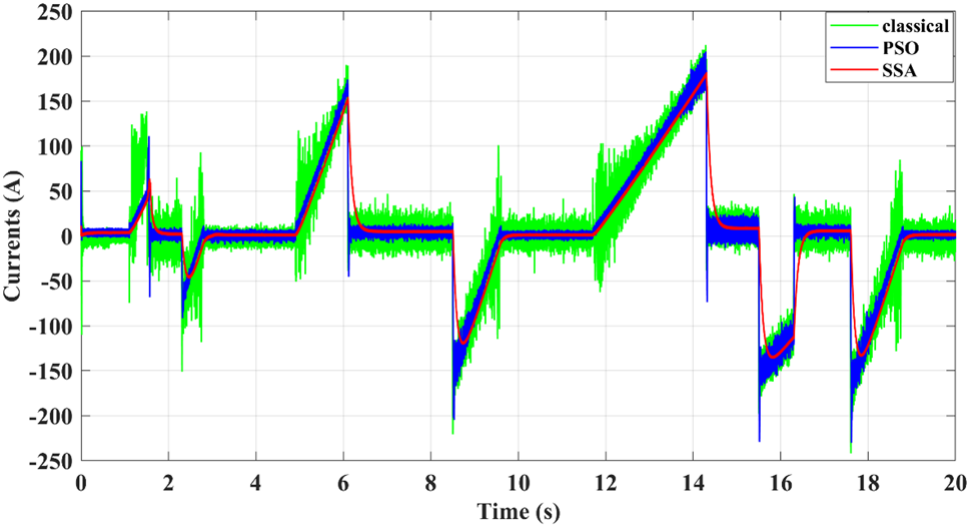
Battery Current

From Fig. 17, representing the current of the battery, the ripple is minimized by the SSA differential flatness to (ΔI = 3 A) instead of (ΔI = 12 A) for PSO differential flatness and (ΔI = 20 A) for the classical differential flatness strategy at the maximum power demanded by the electric vehicle (about 21 kW).

**Figure 18:**
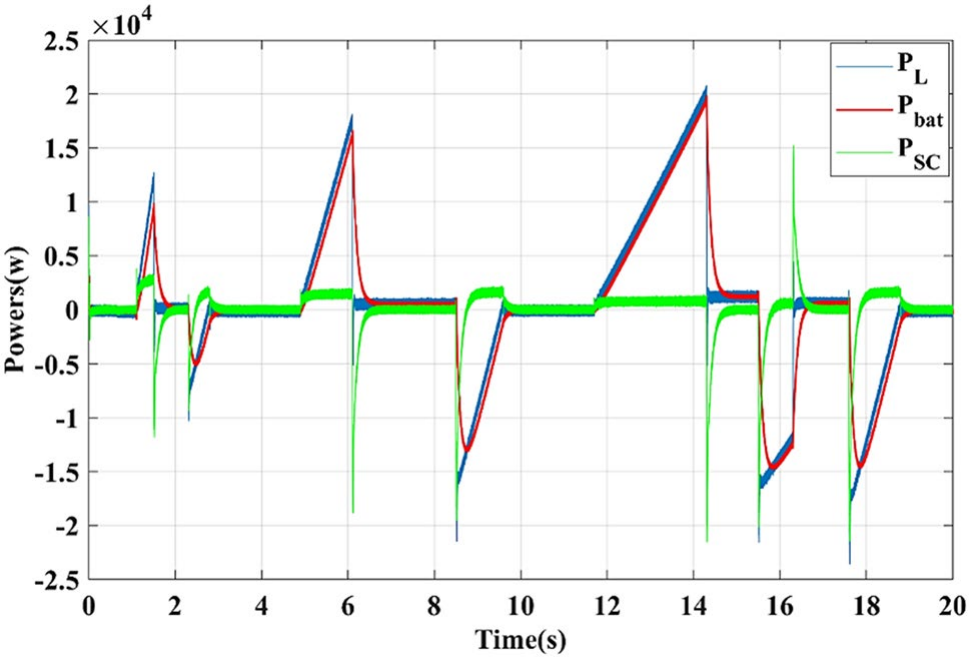
Load, battery, and supercapacitor power waveforms

As illustrated in Fig. 18, the battery provides the average power demanded by the traction system and receives energy during the braking phases. The supercapacitor assists the battery during transient periods (acceleration and deceleration phases), which agrees with the adopted energy management strategy. These results confirm the efficiency of the suggested EMS in managing both energy storage systems.

**Figure 19:**
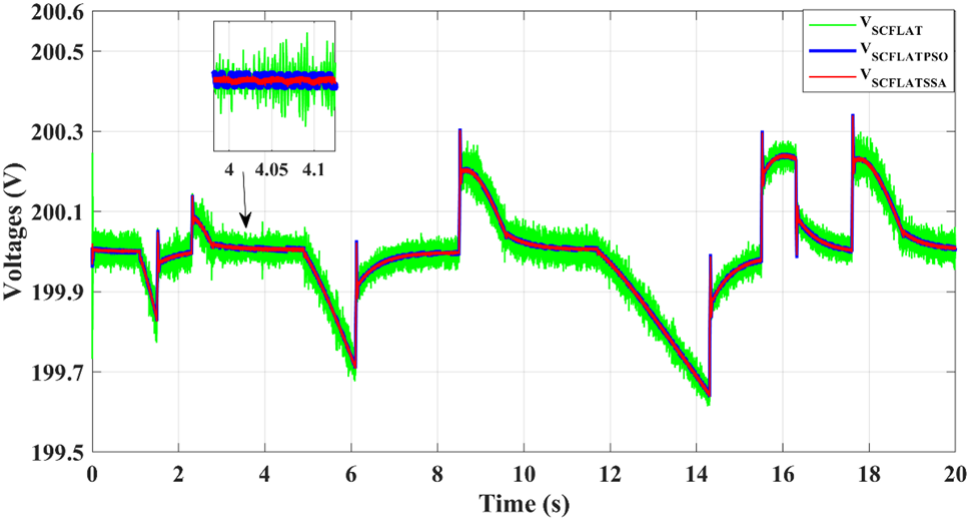
Supercapacitor voltage.

Compared to the PSO and classical differential flatness strategy, the proposed EMS algorithm shows faster supercapacitor voltage dynamics with fewer ripples, as depicted in Fig. 19, During the acceleration and deceleration phases, the SC provides the electric vehicle power until the power provided by the battery reaches its reference, which improves the DC-bus voltage stability.

The suggested technique adjusts its control parameters in accordance with the measured cost function, resulting in enhanced system performance for battery current harmonics, DC-bus voltage ripples, and voltage overshoots.

## Conclusion and future works

This work proposed a new optimized energy management strategy for a battery/supercapacitor-based hybrid power system dedicated to an electric vehicle. Based on the optimal difference flatness, the proposed management technique seeks to manage both sources’ power well depending on the load demand. The main goal of this energy management is to optimize the power quality by reducing the current harmonics while satisfying the SynRM motor power demand, which positively impacts the battery lifecycle. Compared to the classical differential flatness strategy, the proposed optimal adaptive differential flatness strategy can protect the battery against peak current during the acceleration and deceleration phases and significantly reduce the battery current harmonics and DC-bus voltage ripples (Δv = 5 V), as well as the voltage overshoots 15 V (3.2%) for a load power of 21 kW. In addition, the online updating approach also enhances the power system behavior under unknown load changes, improving its robustness and efficiency. The obtained co-simulation results using the C2000 launchxl-f28379d DSP board confirm the effectiveness of the suggested energy management strategy.

The proposed EMS holds the potential for broader applicability across various power systems, including those reliant on fuel-cell technology within hybrid electric vehicles. Our ongoing research involves exploring alternative objective functions within the proposed EMS framework and investigating the efficacy of diverse optimization algorithms. These aspects will be further elucidated in our forthcoming publications.

### Supplementary Information

Supplementary Information.
