# Supplementary material for: Energy management of electric vehicle using a new strategy based on slap swarm optimization and differential flatness control
Source: Sci Rep. 2024 Feb 13;14:3629. doi: 10.1038/s41598-024-53396-3 (PMC11269717; doi:10.1038/s41598-024-53396-3)
Supplement: Supplementary file 1 — Supplementary Information. [file 41598_2024_53396_MOESM1_ESM.docx]

**APPENDIX**

Table A1. Synchronous Reluctance Motor Parameters.

| Parameter | Value |
| --- | --- |
| Continuous output power (kW) | 45 |
| Peak torque in intermediate mode (N.m) | 170 |
| Peak torque in continuous mode (Nm) | 300 |
| Rated speed (tr/min) | 3300 |
| Base speed (tr/min) | 170 |
| Rated Current (A) | 64.40 |
| Resistance of the stator windings R(Ω) | 0.3256 |
| Supply Voltage (V) | 462.2 |
| Number of pole pairs p | 2 |
| d-axis inductance L_d_ (mH) | 73.2 |
| q-axis inductance L_q_ (mH) | 7.3 |

Table A2. Vehicle Parameters.

| Parameter | Value |
| --- | --- |
| Masse of vehicle m_v_ (kg) | 1150 |
| Transmission ratio i | 10 |
| Aerodynamic drag coefficient C_d_ | 0.32 |
| Tire rolling resistance coefficient μ  rotational inertia k_m_ | 0.015  1.1 |
| Vehicle frontal area A_f_ (m^2^) | 2.5 |
| Air density ξ(kg/m^3^) | 1.28 |
| Wheel radius r (m) | 0.33 |
| Earth gravity g (m/s2) | 9.81 |
